# Associations between fatty acid composition in serum cholesteryl esters and liver fat, basal fat oxidation, and resting energy expenditure: a population-based study

**DOI:** 10.1093/ajcn/nqab221

**Published:** 2021-07-05

**Authors:** Michael Fridén, Fredrik Rosqvist, Joel Kullberg, Håkan Ahlström, Lars Lind, Ulf Risérus

**Affiliations:** Department of Public Health and Caring Sciences, Clinical Nutrition and Metabolism, Uppsala University, Uppsala, Sweden; Department of Public Health and Caring Sciences, Clinical Nutrition and Metabolism, Uppsala University, Uppsala, Sweden; Department of Surgical Sciences, Radiology, Uppsala University, Uppsala, Sweden; Antaros Medical AB, BioVenture Hub, Mölndal, Sweden; Department of Surgical Sciences, Radiology, Uppsala University, Uppsala, Sweden; Antaros Medical AB, BioVenture Hub, Mölndal, Sweden; Department of Medical Sciences, Clinical Epidemiology, Uppsala University, Uppsala, Sweden; Department of Public Health and Caring Sciences, Clinical Nutrition and Metabolism, Uppsala University, Uppsala, Sweden

**Keywords:** fatty acid composition, cholesteryl esters, liver fat, energy expenditure, fat oxidation, respiratory quotient

## Abstract

**Background:**

We have repeatedly shown in short-term feeding trials that a high intake of dietary n–6 PUFAs, i.e. linoleic acid, prevents liver fat accumulation compared with saturated fat. However, population-based data is lacking and the mechanisms behind such effects are unclear.

**Objective:**

To investigate associations between serum cholesteryl ester (CE) fatty acids and liver fat, basal fat oxidation [respiratory quotient (RQ)], and resting energy expenditure (REE). We hypothesized that PUFA in particular is inversely associated with liver fat and that such a relation is partly explained by a PUFA-induced increase in basal fat oxidation or REE.

**Methods:**

Cross-sectional analyses using linear regression models in a population-based cohort with data on serum CE fatty acid composition and liver fat (*n* = 308).

**Results:**

Linoleic acid (18:2n–6) (β = −0.03, 95% CI: −0.06, −0.001) and Δ5 desaturase index were inversely associated, whereas, γ-linolenic acid (18:3n–6) (β = 0.59, 95% CI: 0.28, 0.90), dihomo-γ-linolenic acid (20:3n–6) (β = 1.20, 95% CI: 0.65, 1.75), arachidonic acid (20:4n–6) (β = 0.08, 95% CI: 0.002, 0.16), palmitoleic acid (16:1n–7) (β = 0.37, 95% CI: 0.04, 0.70), Δ6 desaturase, and stearoyl CoA desaturase-1 (SCD-1) index were directly associated with liver fat after adjustment for confounders. Several serum CE fatty acids were correlated with both liver fat and REE, but only the association between DHA (22:6n–3) and liver fat was clearly attenuated after adjustment for REE (from β = −0.63 95% CI: −1.24, −0.02 to β = −0.34, 95% CI: −0.95, 0.27). Palmitoleic acid and SCD-1 were weakly inversely correlated with RQ but could not explain a lower liver fat content.

**Conclusions:**

Several serum CE fatty acids are associated with liver fat, among them linoleic acid. Although we identified novel associations between individual fatty acids and RQ and REE, our findings imply that PUFAs might prevent liver fat accumulation through mechanisms other than enhanced whole-body energy metabolism.

## Introduction

Accumulation of fat in the liver (hepatic steatosis) is the key disorder in nonalcoholic fatty liver disease (NAFLD), which is strongly associated with increased risk of cardiometabolic diseases (1–3). Excessive energy intake is the main driver of steatosis, but accumulating evidence suggest that also the composition of fat and carbohydrate could play a major role (4). A higher total dietary fat content has been associated with hepatic steatosis (5, 6), although the quality of fat consumed, as indicated by randomized controlled feeding trials, may play a partial role in these observations (7–10). When matched for calories (7–10) and macronutrient composition (8, 10), PUFAs and MUFAs seem to counteract an increase in liver fat compared with SFAs. However, the mechanisms underlying these effects remain unclear. In contrast to SFA, postprandial oxidation rates of isotope-labeled PUFAs and MUFAs have generally been shown to be higher in both humans and rats (11–14), possibly explaining the favorable effects of certain unsaturated fatty acids. Altered substrate metabolism by unsaturated fats are supported by smaller human trials using indirect calorimetry (15–18), although conflicting results have been reported (19, 20). Furthermore, PUFAs tend to downregulate the transcription of genes involved in lipogenesis and upregulate genes involved in ß-oxidation and thermogenesis (21). Isocaloric studies in rodents have also shown that PUFAs and MUFAs decrease body fat and ectopic fat deposition compared with SFA while increasing energy expenditure (22–24). Other studies have, however, failed to detect an effect on the latter (25). Findings from rodent models on the impact of fatty acids on energy expenditure are partly supported by longer-term human trials (17, 26, 27), whereas others have failed to show an effect (19, 20, 27). Consequently, although controversy remains, a higher fat oxidation rate and increased energy expenditure may provide plausible mechanisms behind the favorable impact of PUFAs on liver fat accumulation. However, since self-reported recall data of fatty acid composition have several limitations, we aimed to design a study that investigated these associations in a population-based study using serum fatty acid biomarkers, which partly reflect dietary fatty acids (28–30), but also endogenous pathways of fat metabolism which may be important for liver fat accumulation and the development of NAFLD. Such a study is, to our knowledge, lacking but needed to provide novel information regarding the links between fatty acids, liver fat content, and energy metabolism on a population level.

Here, we investigated the associations between fatty acids measured in serum cholesteryl esters (CEs) and liver fat content, basal fat oxidation [assessed as respiratory quotient (RQ)], and resting energy expenditure (REE). Further, we examined whether potential associations between fatty acids and liver fat might be mediated by altered energy metabolism.

## Methods

### Subjects

**FIGURE 1:**
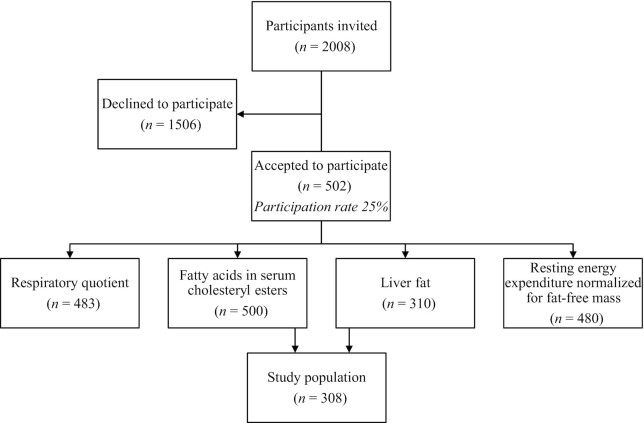
Flow chart of the POEM study.

This cross-sectional study is based on baseline data of the population-based POEM (Prospective investigation of Obesity, Energy and Metabolism) cohort, initiated in Uppsala, Sweden, in 2010, with the aim of studying the pathophysiological links between obesity and vascular dysfunction and future cardiovascular disorders (31). Participants were recruited in a random order by mail 1 mo after their 50th birthday between October 2010 and October 2016 from an official registry of inhabitants in Uppsala. A total of 502 males and females agreed to participate (25%). Of these, fatty acid composition data in serum CEs were obtained from 500 subjects, basal RQ from 483 subjects, REE normalized for fat-free mass (REE/FFM) from 480 subjects, and liver fat content were obtained from 310 subjects. Data on liver fat content and fatty acid composition were available for 308 subjects (**Figure 1**). All participants gave their written informed consent. The study was approved by the Ethics Committee of Uppsala University and conformed to the ethical guidelines of the Declaration of Helsinki.

Blood samples and anthropometric measurements were taken in the morning after an overnight fast. Blood samples were collected on the same day as the indirect calorimetry measurements, however, liver fat assessment using MRI was not performed until a couple of weeks later. Height and weight were recorded and waist circumference (WC) was measured at the umbilical level. BMI was calculated as the weight in kilograms divided by the height in meters squared (kg/m^2^). Total serum cholesterol, LDL cholesterol, HDL cholesterol, triglycerides (TG), fasting plasma glucose, and fasting insulin concentrations were assessed by standard laboratory techniques at Uppsala University Hospital. All participants completed a comprehensive lifestyle questionnaire regarding diet, alcohol intake, and smoking habits, among others.

### Serum CE fatty acid composition

Fatty acid composition in serum CEs was measured using GC (Agilent Technologies system, 6890 N) as previously described (32). A total of 13 fatty acids (14:0–22:6n–3) were measured and individually presented as the proportion of total fatty acids in serum CEs (**Table 1**). Stearoyl CoA desaturase-1 (SCD-1), Δ5 desaturase (D5D), and Δ6 desaturase (D6D) were estimated using the product-to-precursor ratio of 16:1n–7/16:0, 20:4n–6/20:3n–6, and 18:3n–6/18:2n–6, respectively.

**TABLE 1:** Population characteristics (*n* = 308)^1^

	All participants (*n*= 308)
Females/males, %	51/49
University education, %	49
Current smokers, %	7.5
VO_2max_, L/min	2.36 ± 0.68
Anthropometrics	
Weight, kg	80.1 ± 15.2
BMI, kg/m^2^	25.8 (4.9)
WC, cm	93 ± 11
Energy metabolism^2^	
REE, kcal	1409.1 ± 248.8
REE/FFM, kcal/kg FFM	28.6 ± 6.6
RQ	0.78 ± 0.06
Body composition	
BF, %	27.8 ± 8.7
FFM, kg	52.3 ± 16.4
Liver fat, %	2.1 (3.7)
NAFLD prevalence, %	22.7
Glucose metabolism	
Fasting plasma insulin, mU/L	3.6 (3.6)
Fasting plasma glucose, mmol/L	5.0 (0.6)
Lipids	
Total serum cholesterol, mmol/L	5.3 ± 1.0
HDL cholesterol, mmol/L	1.4 (0.4)
LDL cholesterol, mmol/L	3.4 ± 0.9
TG, mmol/L	1.0 (0.7)
Serum cholesteryl ester fatty acids^3^	
Myristic acid 14:0, %	0.76 ± 0.18
Pentadecanoic acid 15:0, %	0.22 ± 0.05
Palmitic acid 16:0, %	10.88 ± 0.63
Palmitoleic acid 16:1n–7, %	2.56 (1.01)
Stearic acid 18:0, %	0.74 (0.15)
Oleic acid 18:1n–9, %	22.25 ± 1.82
Linoleic acid 18:2n–6, %	50.99 ± 3.66
γ-linolenic acid 18:3n–6, %	0.82 ± 0.31
α-linolenic acid 18:3n–3, %	1.02 ± 0.25
Dihomo-γ-linolenic acid 20:3n–6, %	0.68 ± 0.18
Arachidonic acid 20:4n–6, %	6.43 ± 1.2
EPA 20:5n–3, %	1.59 (0.76)
DHA 22:6n–3, %	0.75 ± 0.18
Stearoyl CoA desaturase-1 (SCD-1)^4^	0.23 (0.09)
Δ5 desaturase (D5D) ^5^	9.42 (3.47)
Δ6 desaturase (D6D) ^6^	0.02 ± 0.007
Dietary intake	
Energy, kcal	2243 (1128)
Fat, E%	34 ± 6
SFA, E%	14 ± 3
MUFA, E%	12 (2)
PUFA, E%	5 (2)
Carbohydrate, E%	42 ± 7
Protein, E%	18 ± 3
Alcohol, E%	3 (3)
Vitamin E, mg	11 (6)
Fiber, g	28 (15)

1 Data are presented as mean ± SD, %, or as median (IQR) for skewed distributed variables.

BF, body fat; FFM, fat-free mass; NAFLD, nonalcoholic fatty liver disease; REE, resting energy expenditure; RQ, respiratory quotient; TG, triglycerides; VO_2max_, maximal aerobic capacity; WC, waist circumference.

2 Assessed by indirect calorimetry.

3 Presented as the proportion of total fatty acids in serum cholesteryl esters.

4 Estimated as 16:1n–7/16:0.

5 Estimated as 20:4n–6/20:3n–6.

6 Estimated as 18:3n–6/18:2n–6.

Intake of dietary fatty acids are partly reflected in serum CE, with generally stronger correlations shown for linoleic acid [Spearman´s rho: 0.34 (*P* < 0.001)] and DHA [Spearman´s rho: 0.38 (*P* < 0.001)] than for even-chain SFAs such as palmitic acid [Spearman´s rho: 0.26 (*P* < 0.001)] and stearic acid [Spearman´s rho: 0.07 (*P* = 0.27)] (28). Furthermore, fatty acids measured in circulating CE have demonstrated strong correlations with fatty acids measured in circulating phospholipids in previous population-based observational and interventional studies from Sweden (33).

As a potential surrogate marker for adipose tissue fatty acid composition and possible long-term reflection of dietary intake, 9 nonesterified fatty acids (NEFA) (14:0–22:6n–3) were measured using the same procedure as for fatty acids in serum CE (i.e. separation using thin-layer chromatography and profiled using GC). Although fatty acids measured in serum CE reflect dietary intake over a couple of days to weeks, NEFA through the slower turnover rate of adipose tissue might reflect dietary intake over several months. NEFA in serum were therefore included as exploratory exposure variables in post hoc analyses.

### Liver fat

Liver fat content was assessed using MRI. All subjects were imaged on a 1.5T clinical MRI system (Achieva, Philips Healthcare) in a supine position using the body coil and 1 dedicated water-fat MRI scan in 1 breath hold. Liver MRI scan parameters were: repetition time (TR)/echo time 1 (TE1)/ΔTE = 8.66/0.92/1.32 ms, 6 unipolar echoes, flip angle 5 degrees. Imaged field of view (FOV) was 384 × 288 × 150 mm^3^, and reconstructed voxel size was 3.0 × 3.0 × 10.0 mm^3^. Water-fat image reconstruction was performed using an in-house developed algorithm. Liver fat concentrations were quantified using manual delineation of as large a volume of interest as possible (from the entire liver) avoiding tissue borders and large vessels and bile ducts. Median fat content in the volume of interest was measured. A more detailed description of the MRI methodology and the quantification of liver fat concentrations has previously been presented (34). Prevalence of NAFLD was determined by a liver fat content exceeding 5.5%.

### Substrate utilization, REE, and maximal aerobic capacity

To assess whole-body substrate utilization and energy metabolism, participants arrived at the laboratory in a fasted state (since midnight) between 09:00 and 10:00. While lying in a supine position, after 1 h of rest, pulmonary gas exchange of oxygen (VO_2_) and carbon dioxide (VCO_2_) were measured over a period of 30 min using a ventilated hood and the indirect calorimetry technique (Jaeger Oxycon Pro, Vyaire Medical). Whole-body RQ was calculated using the following formula: VCO_2_/VO_2_. An RQ value of 1 indicates pure carbohydrate oxidation whereas an RQ value of 0.7 indicates pure fat oxidation. For post hoc analyses, the whole-body basal fat oxidation rate was calculated using the equation developed by Frayn (35) and was normalized for FFM, as analyzed by bioelectrical impedance analysis (BIA) (Tanita BC-418). REE was calculated using the abbreviated Weir equation (36) and was also normalized for FFM.

A graded maximal exercise test, using a bicycle ergometer and pulmonary gas exchange (Jaeger Oxycon Pro, Vyaire Medical), was used to measure maximal aerobic capacity (VO_2max_). Participants were instructed to work until volitional exhaustion while the workload was increased by 10 W every minute, starting at 30 W for females and 50 W for males. Maximal heart rate, blood pressure, vital capacity, VCO_2_ and VO_2_ consumption were recorded.

### Dietary intake

A self-administered semiquantitative FFQ consisting of 139 items (including alcohol) was completed by the participants to assess usual frequency of consumption over the past year. Nutrient composition was calculated using the Swedish Food Composition Database and age-specific portion sizes. Reported intakes of dietary fatty acids were presented as relative intakes (percent of total fat). A shorter version of the updated FFQ, consisting of 96 items has been validated against fatty acid biomarkers in subcutaneous adipose tissue and 14-d weighed food records, demonstrating small/moderate (biomarkers) to strong (weighed food records) correlations and good reproducibility (37, 38).

### Statistical analyses

The primary analysis of this study was to investigate the associations between serum CE fatty acids and liver fat content. Secondary analyses included the associations between serum CE fatty acids and REE and RQ and whether these associations could explain potential links between serum CE fatty acids and liver fat content. Correlation analyses (Pearson) were performed between fatty acids in serum CEs (independent variables) and RQ, REE/FFM, and liver fat (dependent variables) as well as between RQ, REE/FFM (independent variables), and liver fat (dependent variable). Simple and multivariable linear regression analyses were performed between fatty acids in serum CEs and liver fat, adjusted for BMI and VO_2max_ in model 1, model 1 + carbohydrate, fat, vitamin E, and alcohol intake in model 2, and model 2 + sex in model 3. For serum CE fatty acids that correlated with both liver fat and REE/FFM, additional multivariable linear regression analyses were performed with liver fat as the dependent variable and serum CE fatty acids as independent variables with REE/FFM included as a covariate in the adjusted model. Regression coefficients (β) with corresponding 95% CIs are reported for each linear regression model. Adjustment for multiple comparisons (*n* = 16 statistical hypothesis tests) was applied in post hoc analyses for the associations between fatty acids in serum CEs and liver fat, RQ, and REE/FFM using the Benjamini–Hochberg procedure with a false discovery rate of 0.05. The distribution of the variables was examined statistically by the Shapiro–Wilk W test and nonnormally distributed variables (W <0.95) were logarithmically transformed. A variance inflation factor (VIF) >5 was used to indicate multicollinearity between included covariates. All statistical analyses were carried out using JMP software version 13.1.0 (SAS Institute, Inc). A *P* value < 0.05 was set as the significance level. For post hoc analyses after adjustment for multiple comparisons, a *P* value < 0.02 was set as the significance level.

## Results

Population characteristics are presented in Table 1. The study population (*n* = 308) did not differ from the full population cohort (*n* = 502) with regard to covariates in Table 1. The study population of middle-aged, equally distributed males (49%) and females (51%) was, despite moderate overweight (BMI of 25.8) and elevated serum cholesterol concentrations (total serum cholesterol of 5.3 mmol/L and LDL cholesterol of 3.4 mmol/L), generally regarded as healthy. The prevalence of NAFLD (22.7%) was not considered high nor low.

### Serum CE fatty acids and liver fat

**FIGURE 2:**
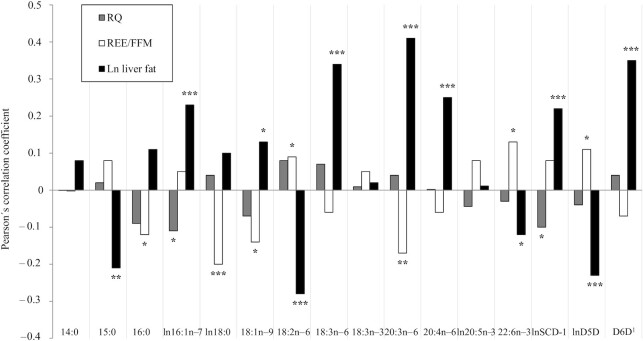
Correlations (Pearson) between fatty acids in serum cholesteryl esters and ln liver fat, respiratory quotient (RQ), and resting energy expenditure normalized for fat-free mass (REE/FFM). 14:0, myristic acid; 15:0, pentadecanoic acid; 16:0, palmitic acid; 16:1n–7, palmitoleic acid; 18:0, stearic acid; 18:1n–9, oleic acid; 18:2n–6, linoleic acid; 18:3n–6, γ-linolenic acid; 18:3n–3, α-linolenic acid; 20:3n–6, dihomo-γ-linolenic acid; 20:4n–6, arachidonic acid; 20:5n–3, EPA; 22:6n–3, DHA; D5D, Δ5 desaturase; D6D, Δ6 desaturase; SCD-1, stearoyl CoA desaturase-1. D5D = 20:4n–6/20:3n–6; D6D = 18:3n–6/18:2n–6; SCD-1 = 16:1n–7/16:0. n(ln liver fat) = 307; n(REE/FFM) = 478; n(RQ) = 481. ^1^Logarithmically transformed for correlations with REE/FFM and RQ. * = *P* < 0.05, ** = *P* < 0.001, *** = *P* < 0.0001.

In serum CEs, linoleic acid (18:2n–6) (*r* = −0.28, *P* = <0.0001), DHA (22:6n–3) (*r* = −0.12, *P* = <0.05), pentadecanoic acid (15:0) (*r* = −0.21, *P* = <0.001), and estimated D5D activity (*r* = −0.23, *P* = <0.0001) were inversely correlated with liver fat, whereas palmitoleic acid (16:1n–7) (*r* = 0.23, *P* = <0.0001), oleic acid (18:1n–9) (*r* = 0.13, *P* = <0.05), γ-linolenic acid (18:3n–6) (*r* = 0.34, *P* = <0.0001), dihomo-γ-linolenic acid (20:3n–6) (*r* = 0.41, *P* = <0.0001), arachidonic acid (20:4n–6) (*r* = 0.25, *P* = <0.0001), and estimated SCD-1 (*r* = 0.22, *P* = <0.0001) and D6D (*r* = 0.35, *P* = <0.0001) activities were positively correlated with liver fat (**Figure 2**). After adjusting for BMI and VO_2max_ (model 1), dietary factors (model 2), and sex (model 3) in multivariable linear regression analyses, palmitoleic acid (β = 0.37, 95% CI: 0.04, 0.70), linoleic acid (β = −0.03, 95% CI: −0.06, −0.001), γ-linolenic acid (β = 0.59, 95% CI: 0.28, 0.90), dihomo-γ-linolenic acid (β = 1.20, 95% CI: 0.65, 1.75), arachidonic acid (β = 0.08, 95% CI: 0.002, 0.16), SCD-1 (β = 0.36, 95% CI: 0.02, 0.71), D5D (β = −0.42, 95% CI: −0.76, −0.08), and D6D (β = 25.63, 95% CI: 11.98, 39.28), remained associated with liver fat whereas oleic acid (β = 0.009, 95% CI: −0.05, 0.06) and DHA (β = −0.24, 95% CI: −0.75, 0.27) did not. In general, the strengths of the associations were similar across the 3 models for most of the fatty acids. The association for pentadecanoic acid was attenuated after adjusting for sex (β = −1.69, 95% CI: −3.75, 0.37) (model 3) (**Table 2**). No multicollinearity was observed between included covariates in the multivariable linear regression analyses.

**TABLE 2:** Simple and multivariable linear regression analyses between fatty acids in serum cholesteryl esters and ln liver fat, adjusted for ln BMI, VO_2max_ (**Model 1**); model 1 + ln carbohydrate, ln fat, ln vitamin E, ln alcohol (**Model 2**); model 2 + sex (**Model 3**)^1^

	Crude model		Model 1 ^2^		Model 2 ^3^		Model 3 ^3^	
	β (95% CI)	*P* value	β (95% CI)	*P* value	β (95% CI)	*P* value	β (95% CI)	*P* value
Myristic acid (14:0)	0.45 (−0.17, 1.07)	0.15	0.26 (−0.27, 0.79)	0.33	0.13 (−0.41, 0.68)	0.63	0.41 (−0.13, 0.95)	0.14
Pentadecanoic acid (15:0)	−4.29 (−6.59, −1.99)	0.0003	−2.56 (−4.51, −0.60)	0.01	−2.75 (−4.77, −0.74)	0.008	−1.69 (−3.75, 0.37)	0.11
Palmitic acid (16:0)	0.17 (−0.005, 0.35)	0.06	0.09 (−0.07, 0.24)	0.27	0.09 (−0.07, 0.24)	0.27	0.09 (−0.06, 0.24)	0.24
ln palmitoleic acid (16:1n–7)	0.72 (0.38, 1.06)	<0.0001	0.32 (0.02, 0.62)	0.04	0.29 (−0.04, 0.63)	0.09	0.37 (0.04, 0.70)	0.03
ln stearic acid (18:0)	0.57 (−0.06, 1.20)	0.08	0.34 (−0.21, 0.88)	0.23	0.38 (−0.17, 0.94)	0.18	0.29 (−0.26, 0.83)	0.30
Oleic acid (18:1n–9)	0.07 (0.01, 0.13)	0.02	0.04 (−0.02, 0.09)	0.19	0.03 (−0.02, 0.09)	0.21	0.009 (−0.05, 0.06)	0.76
Linoleic acid (18:2n–6)	−0.08 (−0.10, −0.05)	<0.0001	−0.03 (−0.06, −0.01)	0.02	−0.03 (−0.06, −0.01)	0.02	−0.03 (−0.06, −0.001)	0.04
γ-linolenic acid (18:3n–6)	1.10 (0.76, 1.45)	<0.0001	0.67 (0.37, 0.98)	<0.0001	0.64 (0.33, 0.96)	<0.0001	0.59 (0.28, 0.90)	0.0002
α-linolenic acid (18:3n–3)	0.08 (−0.36, 0.52)	0.73	−0.004 (−0.38, 0.37)	0.98	−0.07 (−0.45, 0.32)	0.73	−0.04 (−0.42, 0.33)	0.82
Dihomo-γ-linolenic acid (20:3n–6)	2.28 (1.72, 2.85)	<0.0001	1.23 (0.69, 1.77)	<0.0001	1.27 (0.71, 1.83)	<0.0001	1.20 (0.65, 1.75)	<0.0001
Arachidonic acid (20:4n–6)	0.21 (0.12, 0.30)	<0.0001	0.08 (0.003, 0.17)	0.04	0.09 (0.01, 0.18)	0.02	0.08 (0.002, 0.16)	0.045
ln EPA (20:5n–3)	0.03 (−0.27, 0.33)	0.84	−0.08 (−0.33, 0.17)	0.52	−0.09 (−0.35, 0.17)	0.49	−0.06 (−0.31, 0.20)	0.66
DHA (22:6n–3)	−0.63 (−1.24, −0.02)	0.04	−0.43 (−0.94, 0.08)	0.10	−0.37 (−0.89, 0.16)	0.17	−0.24 (−0.75, 0.27)	0.36
ln stearoyl CoA desaturase-1 (SCD1)	0.73 (0.37, 1.09)	<0.0001	0.32 (0.003, 0.64)	0.048	0.28 (−0.07, 0.63)	0.12	0.36 (0.02, 0.71)	0.04
ln Δ5 desaturase (D5D)	−0.81 (−1.19, −0.43)	<0.0001	−0.46 (−0.79, −0.13)	0.007	−0.44 (−0.79, −0.09)	0.01	−0.42 (−0.76, −0.08)	0.02
Δ6 desaturase (D6D)	49.48 (34.77, 64.20)	<0.0001	29.08 (15.72, 42.44)	<0.0001	28.17 (14.23, 42.12)	<0.0001	25.63 (11.98, 39.28)	0.0003

1 Data are presented as β, regression coefficient with 95% CI with corresponding *P* value.

D5D, Δ5 desaturase (20:4n–6/20:3n–6); D6D, Δ6 desaturase (18:3n–6/18:2n–6); SCD-1, stearoyl CoA desaturase-1 (16:1n–7/16:0); VO_2max_, maximal aerobic capacity.

2 *n* = 284.

3 *n* = 280.

Of the serum CE fatty acids inversely associated with liver fat, end-quartile comparisons showed that the difference in liver fat was largest for linoleic acid (1.71%, 93% relative difference), followed by pentadecanoic acid (1.35%, 71% relative difference). For serum CE fatty acids positively associated with liver fat, end-quartile comparisons of dihomo-γ-linolenic acid showed the largest difference in liver fat (3.05%, 229% relative difference) (**Supplemental Table 1**).

### Serum fatty acids in NEFA and liver fat

In post hoc analyses, serum NEFA palmitic acid (16:0) was positively correlated (*r* = 0.25, *P* = <0.0001) whereas linoleic acid was inversely correlated (*r* = −0.12, *P* = <0.05) with liver fat content; no other correlations were observed (**Supplemental Figure 1**). In addition, fatty acids in serum CEs and NEFA demonstrated small to moderate correlations with fatty acids derived from FFQs (**Supplemental Figure 2**). Lastly, minor differences in serum CE fatty acid proportions were observed between males and females (**Supplemental Table 2**).

### Serum CE fatty acids and RQ

In serum CEs, only palmitoleic acid (*r* = −0.11, *P* = <0.05) and estimated SCD-1 activity (*r* = −0.10, *P* = <0.05) were weakly negatively correlated with RQ. However, no other fatty acids were correlated with RQ (Figure 2). Conversely, no correlation was observed between basal RQ and liver fat content (*r* = −0.03, *P* = 0.63).

Post hoc analyses using the calculated basal fat oxidation rate instead of RQ demonstrated similar, although somewhat stronger, correlations with serum CE fatty acids (**Supplemental Table 3**).

### Serum CE fatty acids and REE

In serum CEs, palmitic acid (*r* = −0.12, *P* = <0.05), stearic acid (18:0) (*r* = −0.20, *P* = <0.0001), oleic acid (*r* = −0.14, *P* = <0.05), and dihomo-γ-linolenic acid (*r* = −0.17, *P* = <0.001) were negatively correlated with REE/FFM, whereas linoleic acid (*r* = 0.09, *P* = <0.05), DHA (*r* = 0.13, *P* = <0.05), and estimated D5D activity (*r* = 0.11, *P* = <0.05) were positively correlated with REE/FFM (Figure 2). In addition, REE/FFM was inversely correlated with liver fat content (*r* = −0.24, *P* <0.0001).

In multivariable linear regression analyses of the fatty acids that were both associated with REE/FFM and liver fat content (Figure 2), oleic acid (β = 0.07, 95% CI: 0.01, 0.13), linoleic acid (β = −0.08, 95% CI: −0.11, −0.05), dihomo-γ-linolenic acid (β = 2.14, 95% CI: 1.57, 2.71), and D5D activity (β = −0.75, 95% CI: −1.13, −0.37), remained associated with liver fat also after adjusting for REE/FFM, whereas DHA did not (β = −0.34, 95% CI: −0.95, 0.27) (**Table 3**).

**TABLE 3:** Simple and multivariable linear regression analyses between fatty acids in serum cholesteryl esters and ln liver fat, adjusted for resting energy expenditure normalized for fat-free mass^1^

	Crude model		Adjusted model^2^	
	β (95% CI)	*P* value	β (95% CI)	*P* value
Oleic acid (18:1n–9)	0.07 (0.01, 0.13)	0.02	0.07 (0.01, 0.13)	0.03
Linoleic acid (18:2n–6)	−0.08 (−0.10, −0.05)	<0.0001	−0.08 (−0.11, −0.05)	<0.0001
Dihomo-γ-linolenic acid (20:3n–6)	2.28 (1.72, 2.85)	<0.0001	2.14 (1.57, 2.71)	<0.0001
DHA (22:6n–3)	−0.63 (−1.24, −0.02)	0.04	−0.34 (−0.95, 0.27)	0.27
ln Δ5 desaturase (D5D)	−0.81 (−1.19, −0.43)	<0.0001	−0.75 (−1.13, −0.37)	0.0001

1 Data are presented as β, regression coefficient with 95% CI with corresponding *P* value. Serum fatty acids in cholesteryl esters that correlated with both liver fat and resting energy expenditure normalized for fat-free mass were included in the crude and adjusted models.

D5D, Δ5 desaturase (20:4n–6/20:3n–6).

2 *n* = 292.

### Adjustment for multiple comparisons (post hoc analyses)

After adjusting for multiple comparisons for the associations between serum fatty acids in CEs and liver fat, RQ, and REE/FFM, DHA was nonsignificantly inversely associated with liver fat in the crude model, whereas palmitoleic acid, linoleic acid, arachidonic acid, SCD-1, and D5D were nonsignificantly associated with liver fat in the full multivariable adjusted model. SCD-1 was nonsignificantly inversely associated with RQ whereas linoleic acid was nonsignificantly positively associated with REE/FFM. Since DHA was not associated with liver fat in these poshoc analyses, it was not included in further models adjusting for REE/FFM. In these latter models, oleic acid was nonsignificantly associated with liver fat. All other associations observed in the main analyses remained after adjusting for multiple comparisons (data not shown).

## Discussion

In this population-based sample of middle-aged individuals, the 2 PUFAs, linoleic acid and DHA, as well as pentadecanoic acid and D5D were inversely correlated with liver fat whereas palmitoleic acid, oleic acid, γ-linolenic acid, dihomo-γ-linolenic acid, arachidonic acid, and SCD-1 and D6D were positively correlated with liver fat. The majority of these associations remained after full adjustments.

Associations between serum CE fatty acids and liver fat in the current study are in accordance with 2 other Swedish population-based studies, both of which observed a negative correlation between linoleic acid and liver fat and a positive correlation between D6D and liver fat assessed directly by proton magnetic resonance spectroscopy (^1^H-MRS) or indirectly via alanine aminotransferase (ALT) (32, 39). Moreover, a positive association was observed for palmitoleic acid, γ-linolenic acid, dihomo-γ-linolenic acid, and SCD-1 after adjusting for relevant covariates in 1 study (39). However, some minor discrepancies were noticeable between our study and the 2 other Swedish population-based studies, partly explained by methodological differences in the assessment of liver fat and a lack of power due to a smaller sample size. Our findings also accord with a previous prospective cohort study using ultrasonography to diagnose steatosis (40). Over a 10-y follow-up period, baseline serum linoleic acid was inversely associated, whereas DHA was nonsignificantly inversely associated with the incidence of fatty liver.

Interestingly, observational studies on self-reported dietary intake in relation to liver fat show a positive correlation between SFA intake and liver fat and a trend towards a negative correlation with MUFA and PUFA intake (41). Conflicting findings have, however, been reported (5, 42). When dietary fatty acid classes were correlated with liver fat in our study, a weak inverse trend was observed for PUFAs (data not shown), partly explained by the weak to moderate correlations between dietary fatty acids and serum CE fatty acids (Supplemental Figure 2). Meanwhile, randomized controlled feeding trials conducted by our group and others point to a greater benefit of mainly consuming unsaturated fat (linoleic acid in particular) over SFA (palmitic acid in particular) in relation to liver fat accumulation (7–10). In accordance with these trials, linoleic acid in serum CEs was inversely associated with liver fat in the current study after adjusting for several covariates. A 1.71% absolute difference (93% in relative numbers) in liver fat was observed between the 4th and 1st quartile of linoleic acid proportion. Considering that NAFLD is diagnosed with a liver fat content of >5.5%, a 1.7% absolute difference is arguably a clinically relevant effect. Furthermore, a mean difference of 8.97% between end-quartile proportions of linoleic acid (data not shown) is in line with the trials discussed above showing a mean difference in change of linoleic acid in serum CEs of 5.8–6.5%, corresponding to a dietary linoleic acid intake of 12–14 E% (7, 8). Interestingly, we also found an inverse association between pentadecanoic acid, a biomarker of dairy fat intake (43) and liver fat content. Biomarkers of dairy fat intake have previously been associated with reduced liver fat in a smaller cross-sectional study (44) but interventional studies are needed to determine a possible causality behind such an association. However, oral supplementation with pentadecanoic acid in rabbits was recently shown to improve multiple liver health indices and attenuate fibrosis (45).

Several serum CE fatty acids as well as D5D were significantly correlated with REE/FFM, although this relation had minor influence on the link between serum CE fatty acids and liver fat. However, DHA could potentially, to a certain extent, explain a lower liver fat content mediated through an increased REE/FFM as indicated in multivariable models. Furthermore, although palmitoleic acid and SCD-1 were significantly inversely correlated with RQ, indicating a higher basal fat oxidation rate, this could not, however, explain the association with a higher liver fat content since RQ itself was not correlated with liver fat. Interestingly, serum and/or plasma fatty acids that have been previously associated with increased cardiometabolic disease risk, showed inverse associations with REE/FFM, i.e. palmitic acid, stearic acid, oleic acid, and dihomo-γ-linolenic acid (46, 47).

Differences in postprandial- and 24-h fat oxidation rates between PUFAs, MUFAs, and SFAs have previously been observed in controlled feeding trials (11–14, 17, 48). However, trials that have assessed fat oxidation in the basal state have generally failed to detect a significant difference (16, 17, 19, 20). Although weak correlations between palmitoleic acid, SCD-1, and RQ were observed in our study, this could not explain lower liver fat. Furthermore, dietary unsaturated fatty acids can increase energy expenditure (22–24) and decrease ectopic fat in rodent models (22–25). Although human data are still conflicting (17, 19, 20, 26, 27, 48, 49), our results on serum CE fatty acids and REE are in line with above-mentioned rodent studies. Interestingly, when DHA and liver fat was adjusted for REE/FFM, the association was markedly attenuated, possibly indicating that DHA may influence liver fat through increased REE. In support of this finding, human supplementation trials of ω-3 as well as studies in rodents have shown increased energy expenditure and reduced liver fat (42, 50–52), possibly explained by a diet-induced increase in mitochondrial energy dissipation (53, 54). Further investigation on the relation between DHA, REE, and liver fat is, however, needed (54).

There are several strengths of this study, including the relatively large population-based sample, the inclusion of both males and females to increase generalizability, the use of objective fatty acid biomarkers instead of self-reported dietary intakes, the use of indirect calorimetry to assess energy metabolism, and the use of MRI to quantify liver fat. Thus, the simultaneous data on serum CE fatty acids, liver fat, and energy metabolism in a population-based setting must be regarded as unique and novel. In addition, several variables such as FFM using BIA and VO_2max_ using a cycle ergometer were measured to make sure valid covariates were adjusted for, although residual confounding is inevitable. However, there are some limitations. The study used a cross-sectional design, which means that causal inferences cannot be drawn. In addition, the population sample consisted of individuals aged 50 y, which limits generalizability, but instead excludes age-related bias. Other limitations include the lack of information on the CV for the indirect calorimetry measurements as well as the lag time between the collection of blood samples and performance of MRI scans. Furthermore, the fatty acid composition is presented as raw area percentages; potential differential response factors for individual fatty acids could not be accounted for, which may introduce bias as split-injection was used. Finally, some caution is warranted when interpreting the data from the main analyses due to the lack of control for multiple testing, a statistical procedure that is albeit debated in explorative settings. However, additional post hoc analyses adjusting for multiple comparisons had minor to moderate influence on the results.

In conclusion, several serum CE fatty acids are associated with liver fat, among them linoleic acid. Although we identified novel associations between individual fatty acids and RQ and REE, our findings imply that PUFAs might prevent liver fat accumulation through mechanisms other than enhanced whole-body energy metabolism.

## Supplementary Material

nqab221_Supplemental_File

## Data Availability

Data described in the manuscript will be made available from the corresponding author upon reasonable request.

## Notes

Supported by the Swedish Research Council (2016-04010), The Swedish Heart-Lung Foundation (20170492), Excellence of Diabetes Research in Sweden (EXODIAB), P.O. Zetterlings foundation, The Swedish Diabetes Foundation, Swedish Research Council for Sustainable Development (FORMAS).

Supplemental Tables 1–3 and Supplemental Figures 1 and 2 are available from the “Supplementary data” link in the online posting of the article and from the same link in the online table of contents at https://academic.oup.com/ajcn/.

Abbreviations used: BIA, bioelectrical impedance analysis; CE, cholesteryl ester; FFM, fat-free mass; NAFLD, nonalcoholic fatty liver disease; NEFA, nonesterified fatty acids; REE, resting energy expenditure; RQ, respiratory quotient; SCD-1, stearoyl CoA desaturase-1; VCO_2_, volume of carbon dioxide; VO_2_, volume of oxygen; VO_2max_, maximal aerobic capacity; D5D, Δ5 desaturase; D6D, Δ6 desaturase.
