# Supplementary material for: Associations between fatty acid composition in serum cholesteryl esters and liver fat, basal fat oxidation, and resting energy expenditure: a population-based study
Source: Am J Clin Nutr. 2021 Jul 5;114(5):1743–51. doi: 10.1093/ajcn/nqab221 (PMC8574708; doi:10.1093/ajcn/nqab221)
Supplement: nqab221_Supplemental_File [file nqab221_supplemental_file.pdf]

Associations between fatty acid composition in serum cholesteryl esters and liver fat, basal fat oxidation and resting energy expenditure: a population-based study (Fridén M)

Online Supplementary Material

Supplementary Table 1. Absolute and relative differences in liver fat content between end-quartiles of fatty acids (FA) in serum cholesteryl esters[1]

	**Liver fat %** (Q_1 of FA)	**Liver fat %** (Q_4 of FA)	**Absolute diff. in liver fat %** (Q_4-Q_1 of FA)	**Relative diff. in liver fat %** (Q_4 vs Q_1 of FA)	P-value[2]
Myristic acid (14:0)	2.18[3]	2.96	0.78	36	0.06
Pentadecanoic acid (15:0)	3.24	1.89	-1.35	71	0.0008
Palmitic acid (16:0)	2.08	2.91	0.83	40	0.04
Palmitoleic acid (16:1n-7)	1.9	3.27	1.37	72	0.0007
Stearic acid (18:0)	2.04	2.81	0.77	38	0.048
Oleic acid (18:1n-9)	2.09	2.93	0.84	40	0.03
Linoleic acid (18:2n-6)	3.54	1.83	-1.71	93	<0.0001
γ-linolenic acid (18:3n-6)	1.71	4.17	2.46	144	<0.0001
α-linolenic acid (18:3n-3)	2.89	2.67	-0.22	8	0.62
Dihomo-γ-linolenic acid (20:3n-6)	1.33	4.38	3.05	229	<0.0001
Arachidonic acid (20:4n-6)	1.82	3.44	1.62	89	<0.0001
Eicosapentaenoic acid (20:5n-3)	2.3	2.29	-0.01	0.4	0.99
Docosahexaenoic acid (22:6n-3)	2.57	2.14	-0.43	20	0.26
Stearoyl CoA desaturase-1 (SCD-1)	1.94	3.37	1.43	74	0.0006
Delta-5 desaturase (D5D)	3.43	1.76	-1.67	95	<0.0001
Delta-6 desaturase (D6D)	1.64	4.19	2.55	155	<0.0001

[1]Data are presented as % with corresponding P-values.

[2]Statistical analyses were performed using independent samples t-tests.

D5D, delta-5 desaturase (20:4n-6/20:3n-6); D6D, delta-6 desaturase (18:3n-6/18:2n-6); SCD-1, stearoyl CoA desaturase-1 (16:1n-7/16:0).

[3]All liver fat values have been back-transformed from ln liver fat.

Supplementary Table 2. Proportions of fatty acids in serum cholesteryl esters among men and women[1]

	**Men (n=152)**	**Women (n=156)**	**P-value[2]**
Myristic acid 14:0 (%)	0.75±0.16	0.77±0.20	0.35
Pentadecanoic acid 15:0 (%)	0.22±0.05	0.23±0.05	0.06
Palmitic acid 16:0 (%)	10.95±0.59	10.81±0.67	0.06
Palmitoleic acid 16:1n-7 (%)	2.45 (0.99)	2.67 (0.97)	0.04
Stearic acid 18:0 (%)	0.78 (0.16)	0.72 (0.16)	<0.0001
Oleic acid 18:1n-9 (%)	22.57±1.84	21.94±1.75	0.002
Linoleic acid 18:2n-6 (%)	50.65±3.63	51.33±3.67	0.10
γ-linolenic acid 18:3n-6 (%)	0.85±0.32	0.80±0.30	0.16
α-linolenic acid 18:3n-3 (%)	1.01±0.26	1.02±0.25	0.83
Dihomo-γ-linolenic acid 20:3n-6 (%)	0.70±0.18	0.66±0.18	0.04
Arachidonic acid 20:4n-6 (%)	6.47±1.18	6.40±1.21	0.62
Eicosapentaenoic acid 20:5n-3 (%)	1.58 (0.73)	1.59 (0.80)	0.99
Docosahexaenoic acid 22:6n-3 (%)	0.73±0.17	0.78±0.19	0.04
Stearoyl CoA desaturase-1 (SCD-1)[3]	0.22 (0.09)	0.24 (0.08)	0.01
Delta-5 desaturase (D5D)[4]	9.04 (3.38)	9.77 (3.33)	0.10
Delta-6 desaturase (D6D)[5]	0.02±0.007	0.02±0.007	0.12

[1]Data are presented as mean±SD, % or as median (IQR) for skewed distributed variables.
Fatty acids are presented as the proportion of total fatty acids in serum cholesteryl esters.
[2]Statistical analyses were performed using independent samples t-tests or for stearic acid (18:0) a Mann-Whitney U test. Palmitoleic acid (16:1n-7), eicosapentaenoic acid (20:5n-3), stearoyl CoA desaturase-1 (SCD-1) and delta-5 desaturase (D5D) were log-transformed.
[3]Estimated as 16:1n-7/16:0. [4]Estimated as 20:4n-6/20:3n-6. [5]Estimated as 18:3n-6/18:2n-6.

Supplementary Table 3. Correlations (Pearson) between fatty acids in serum cholesteryl esters and basal fat oxidation rate and respiratory quotient (RQ)[1]

	**Basal fat oxidation (g/kg FFM/min)**	**P-value**	**RQ**	**P-value**
Myristic acid (14:0)	0.006	0.89	-0.001	0.98
Pentadecanoic acid (15:0)	0.02	0.64	0.02	0.71
Palmitic acid (16:0)	-0.01	0.75	-0.09	0.06
ln Palmitoleic acid (16:1n-7)	0.13	0.006	-0.11	0.01
ln Stearic acid (18:0)	-0.15	0.0007	0.04	0.44
Oleic acid (18:1n-9)	-0.03	0.53	-0.07	0.10
Linoleic acid (18:2n-6)	-0.01	0.8	0.08	0.06
γ-linolenic acid (18:3n-6)	0.03	0.46	0.009	0.84
α-linolenic acid (18:3n-3)	-0.07	0.11	0.07	0.11
Dihomo-γ-linolenic acid (20:3n-6)	-0.14	0.003	0.04	0.36
Arachidonic acid (20:4n-6)	-0.03	0.45	0.002	0.96
ln Eicosapentaenoic acid (20:5n-3)	0.08	0.1	-0.04	0.33
Docosahexaenoic acid (22:6n-3)	0.09	0.048	-0.03	0.53
ln Stearoyl CoA desaturase-1 (SCD-	0.14	0.003	-0.10	0.03
ln Delta-5 desaturase (D5D)	0.10	0.03	-0.04	0.37
Delta-6 desaturase (D6D)	-0.06	0.21	0.04	0.33

[1]Data are presented as Pearson r with corresponding P-values.
D5D, delta-5 desaturase (20:4n-6/20:3n-6); D6D, delta-6 desaturase (18:3n-6/18:2n-6); SCD-1, stearoyl CoA desaturase-1 (16:1n-7/16:0).
n=478.

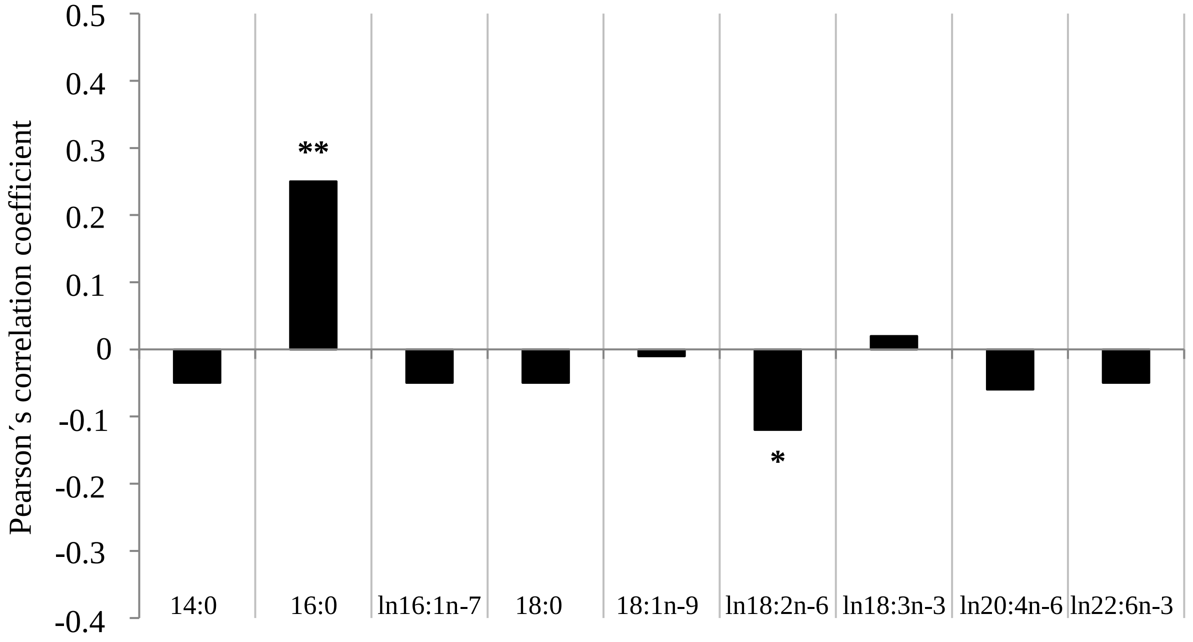

Supplementary Figure 1. Correlations (Pearson) between non-esterified fatty acids and ln liver fat.
14:0, myristic acid; 16:0, palmitic acid; 16:1n-7, palmitoleic acid; 18:0, stearic acid; 18:1n-9, oleic acid; 18:2n-6, linoleic acid; 18:3n-3, α-linolenic acid; 20:4n-6, arachidonic acid; 22:6n-3, docosahexaenoic acid.
n=304-306.
***** = P<0.05, ****** = P<0.0001.

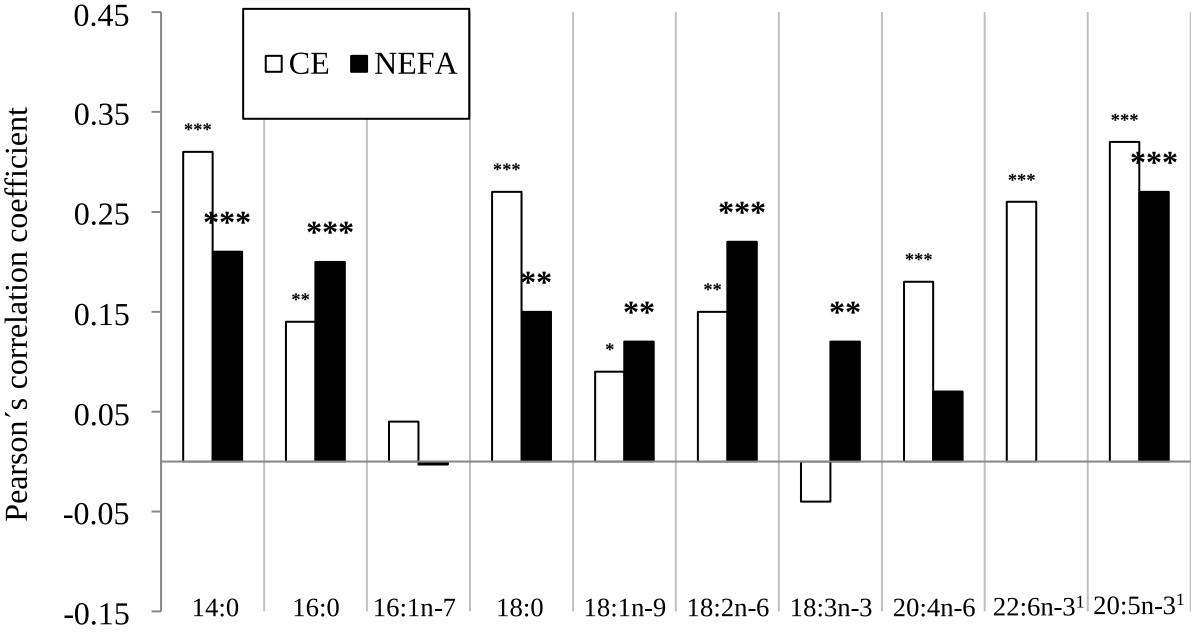

Supplementary Figure 2. Correlations (Pearson) between dietary fatty acids (% of total fat) assessed by food frequency questionnaire and fatty acids in serum cholesteryl esters (CE) and non-esterified fatty acids (NEFA). 14:0, myristic acid; 16:0, palmitic acid; 16:1n-7, palmitoleic acid; 18:0, stearic acid; 18:1n-9, oleic acid; 18:2n-6, linoleic acid; 18:3n-3, α-linolenic acid; 20:4n-6, arachidonic acid; 20:5n-3, eicosapentaenoic acid; 22:6n-3, docosahexaenoic acid.
n(CE)=488; n(NEFA)=484-487.
Non-normally distributed fatty acids in CE, NEFA and from food frequency questionnaires were log-transformed.
[1]Analyzed non-parametrically with Spearman rank correlation and presented as Spearman´s rho.
* = P<0.05, ** = P<0.01, *** = P<0.0001.
